# How to improve communication competence of government leading officials in intercultural environment?—the driving role of intercultural psychological factors

**DOI:** 10.3389/fpsyg.2025.1544626

**Published:** 2025-04-28

**Authors:** Ting Wang, Jingping Li

**Affiliations:** ^1^School of Marxism, Shaanxi Normal University, Xi'an, China; ^2^School of Marxism, Xi’an Jiaotong University, Xi'an, China

**Keywords:** intercultural communication competence, intercultural psychological factors, government leading officials, attitude and awareness, intercultural environment

## Abstract

**Introduction:**

In the context of the Belt and Road Initiative, inland China city such as Xi’an, is in a situation where native and foreign cultures are intertwined. Government leading officials are crucial to the effectiveness of intercultural communication in the region, but little attention has been paid to the government leading officials’ intercultural communication competence (ICC) and its factors.

**Methods:**

In this investigation, the factors of ICC were expanded to psychological factors (attitude and awareness of intercultural communication), knowledge factors (native and foreign cultural knowledge), and skill factors (resilience and pragmatic skills for intercultural communication). A structured questionnaire was administered to 578 government leading officials in Xi’an. The collected data were analyzed using descriptive statistics, factor analysis, difference analysis, and regression analysis.

**Results:**

The study revealed that among the factors of ICC, intercultural communication awareness exerted the strongest influence on resilience skills. Additionally, the highest foreign language score, participation in training, education status, and frequency of contact with foreigners significantly impacted ICC.

**Conclusion:**

This study expanded existing intercultural communication models. Based on the results of the analysis, possible measures to promote ICC of government leading officials were proposed, including the enhancement of foreign language skills, participation in professional training, intercultural situational education, and study abroad.

## 1 Introduction

With the development of globalization, it has become the consensus of countries all over the world to cultivate international talents with intercultural communication competence (ICC). Similarly, a new generation of government leading officials with tolerant intercultural attitudes and the competence to embrace differences are essential for humankind (Pusch, 2009). Researches on ICC have been conducted to analyze the effective communication and behaviors of groups from different cultural backgrounds [for example international students (Saini and Ardhy, 2023; Vromans et al., 2023; Zhang and Zeng, 2023), multinational company employees (Presbitero and Attar, 2018), immigrants (Samovar et al., 2013; Dalib et al., 2023), workers (Martin and Nakayama, 2015), businessman (Jiao et al., 2020), university supervisors (Anuar et al., 2025)], in intercultural situations. However, there are few studies on ICC of government leading officials. The awareness, attitude, and emotion of a country or region’s government leading officials toward foreign cultures will affect the identification degree of the foreign countries within the country or region and will influence the behavioral intentions of foreign tourists, foreign businessmen, and foreign immigrants. Therefore, the ICC of government leading officials needs to be studied.

### 1.1 Communication competence in intercultural environment

ICC can be understood from the developmental process or elemental components perspective. When people migrate from a familiar cultural environment to another relatively unfamiliar cultural environment, they generally go through different stages of exploration, adaptation, and acceptance. Bennett proposed the developmental intercultural model (DIMS), which consists of six successive stages: rejection, defense, devaluation, acceptance, adaptation, and integration (Hammer et al., 2003). Individuals are strongly influenced by their own culture in the first three stages, while they gradually enter and experience new cultural situations in the last three stages. The maturity model of ICC (King and Baxter Magolda, 2005) also belongs to the form of the developmental process. According to Fantini (2009), ICC is the competence to demonstrate effective and appropriate behavior when interacting with people from different linguistic and cultural backgrounds. Deardorff (2006) defined ICC as “the competence to communicate effectively, appropriately, based on one’s intercultural knowledge, skills, and attitudes.” A pyramid model of intercultural competence, “attitude-knowledge-skill,” has been developed to explain ICC.

Some studies have viewed ICC in terms of its elemental components. Byram (1997) proposed a multifactor model of ICC, which examined it in terms of the four interdependent factors of intercultural knowledge, skills, attitudes, and awareness. Chen and Starosta (2000) analyzed ICC in terms of intercultural sensitivity, intercultural awareness, and intercultural skills. Samovar et al. (2013) summarized ICC in terms of knowledge, skills, and motivation.

The consensus reaches by most researchers on ICC is that it is built on knowledge, skills, awareness, and attitude (Khan et al., 2023). When this theory is introduced to China, it will be flawed if it lacks an investigation of the ability to use a foreign language. The knowledge factor also needs to be further subdivided.

Koester and Lustig (2015) argued that how to manage potential conflicts of cultures and how to mitigate prejudice and ethnocentric tendencies in the search for common ground is what should be considered more in intercultural communication. Martin (2015) argued that intercultural communication should shift from to homogeneous ethnocultural groups to a focus on fluid, dynamic multiculturalism for true intercultural communication. Schwarzenthal et al. (2017) suggested that ICC was positively correlated with intercultural contact and national identity exploration. Therefore, understanding others (by engaging in intercultural contact) and understanding oneself (by exploring one’s ethnic background) were both important for developing critical intercultural communication skills (Zhang and Noels, 2024).

However, a significant body of existing research focuses on how minority group members adapt to unfamiliar cultural environments (Rak et al., 2023). Minority group members often lack the power to influence cultural policies. In contrast, for Chinese government leading officials, their role has transcended traditional cultural management and administrative functions, evolving into that of key facilitators who effectively coordinate cultural differences (Jin and Cortazzi, 2022). Study showed that organizational identity was crucial to organization development and was influenced by many factors, among which leadership communication was the key factor (Mayfield et al., 2020).

Previous studies have focused on the ICC of immigrants, foreign language learners, and business traders when they enter an unfamiliar cultural environment. For example, there was a study that explored how Chinese government officials interpreted American professionals’ hosting behaviors (Wang and Spencer-Oatey, 2015). Few attention has been paid to how people in familiar cultural environments perceive groups such as inbound foreign tourists and business people. In the context of the Belt and Road Initiative, Chinese government leading officials are facing the situation of how to communicate with foreign nationals in China. Now, there is a lack of research on the ICC of Chinese leading officials, especially government leading officials.

### 1.2 The importance of improving ICC of Chinese government leading officials

There are differences between Chinese government leading officials and Western civil servants. Government leading officials are “those who have certain power and hold certain leading positions in the party and government system” (《The Complete Book of the State Civil Service》, China, 1994). Government leading officials can make decisions and plans in the development of a region or department. Because of their high level of power and decision-making positions, Chinese government leading officials are required to have a strong sense of commitment and historical mission and an efficient learning competence.

Over the past decade, under the impetus of the Belt and Road initiative, China has established bilateral economic and trade relations with more countries along the Belt and Road. China’s foreign work has become more proactive and positive. Under the development of the Belt and Road, the urban appearance and cultural environment of inland cities of China have undergone many changes. For example, inland cities have become more open and more connected to the world. Increasing immigration in China means that inland cities are experiencing long-term intercultural contact between the majority and minority group members. Government leading officials in inland cities face more intercultural situations. For example, frequent interaction with parliaments, political parties, and civil society organizations; frequent interaction with foreign government officials, foreign trade personnel, foreign students, and resident foreigners in China. Government leading officials in inland cities must adapt to the differences between different cultures, different social systems, and different stages of development. As majority group members, government leading officials’ cultural empathy will be stimulated, and the cultural psychology and cultural behaviors of leading officials will change (Egitim and Akaliyski, 2024). These changes may include understanding other countries’ languages, policies, food preferences, and festivals. However, the impact of these interactions on the ICC of government leading officials in inland cities of China has not yet received much attention and needs to be studied.

Xi’an is the largest inland city in northwestern China and the capital city of Shaanxi Province, which is both historical and modern. Xi’an was historically the starting point of the ancient Silk Road and is now an important pivot city of the Belt and Road. To fulfill requirements for developing Belt and Road, Xi’an has organized several Belt and Road forums, operated the “Chang’an” China-European train, constructed the Shaanxi pilot free trade zone, and continuously strengthened humanistic communication and cooperation. This has put forward new requirements for Xi’an’s government leading officials to promote their ICC. Xi’an’s geographical advantages in the Belt and Road Initiative have presented new requirements for government leading officials to transform their thoughts, build platforms for multicultural exchange, and strengthen their cultural responsibilities. At the same time, these advantages provide an excellent observation point for investigating the ICC of Chinese government leading officials.

To expand the scope of research on ICC and to gain a deeper understanding of the current situation and dilemma of ICC of government leading officials in the context of Belt and Road. This study investigated the ICC of government leading officials in Xi’an through developing scale, constructing models, and analyzing data.

The issues we investigated in this study included:

1. What is the current status of the ICC of government leading officials and its factors?
2. What are the differences in the influence of different demographic variables on the factors of ICC?
3. The analysis of factors and promotion strategies of ICC of government leading officials.

The hypotheses of this study were:

*H1*: The native cultural knowledge, foreign cultural knowledge, intercultural communication attitude, intercultural communication awareness, and pragmatic skills for intercultural communication positively influence resilience skills.

*H2*: Variables of leadership position, age, highest foreign language score, education status, and participation in training are the influencing variables of ICC.

## 2 Participants and methods

### 2.1 Participants

The questionnaires were distributed from August 2020 to December 2020. We obtained a series of data by distributing questionnaires to participants in various training courses organized by the Shaanxi Academy of Governance. In China, Academies of Governance are schools that periodically train party and government leading officials. The second author of the paper frequently conducted academic lectures for government leading officials at the Shaanxi Academy of Governance, targeting non-fixed groups of government leading officials. This provided him with convenient access to a wide range of government leading officials, creating favorable conditions for the implementation of this study. The research team selected training classes for government leading officials who were likely to participate in the Belt and Road Initiative. For example, the Shaanxi Academy of Governance organized the “Training course for division-head level government leading officials of the China Railway First Survey and Design Institute Group,” “Training course on new ideas and capabilities for section-head level government leading officials in Xi’an,” “Training Program for Young and Middle-Aged officials of Democratic Parties in Xi’an,” which were mainly for officials with higher positions. Before the lectures, after obtaining consent from the government leading officials, members of the research team would briefly explain the purpose, methods, and duration of the survey. The research team would then distribute the questionnaires and collect them after the lectures. These temporary training classes typically had a small number of participants, ranging from 30 to 60 individuals, so questionnaires were distributed to members of 14 training classes in total. The government leading officials at the Section-head level, division-head level and bureau-director level in Xi’an’s party and government organizations, public institutions, and state-owned enterprises were the main participants of this investigation. These government leading officials were in the new environment brought by the Belt and Road. We distributed a total of 636 paper questionnaires, and 578 valid questionnaires were returned. Participants did not receive incentives for completing the survey.

Among the valid questionnaires, 70% of the participants were male and 30% were female, 25% were under 40 years old and 75% were 40 years old or above, which were consistent with the age structure of the current Chinese government leading officials. In terms of experience in intercultural environments, 55% of the participants had never interacted with foreigners, while 45% had experienced interacting with foreigners. 59% of the participants had no experience of going abroad, while 41% had gone abroad for traveling, working, or studying. 7.4% of the participants were engaged in or in charge of foreign affairs.

### 2.2 Research methods

#### 2.2.1 Scale design

In the existing frameworks of ICC, the model proposed by British scholar Byram (1997) is the most typical, widely recognized, and influential model. This framework adhered to the cognitive classification principle of competence. Byram divides ICC into four factors: knowledge, attitude, awareness, and skills. When applying Byram’s ICC model, this study made two modifications to meet the requirements for analyzing the ICC of government leading officials: Adding “pragmatic skills for intercultural communication” to Byram’s model to address the original framework’s lack of emphasis on language use skills and non-verbal communication skills; Dividing the “knowledge” factor into “native cultural knowledge” and “foreign cultural knowledge,” highlighting the interaction between one’s own culture and the target culture in intercultural communication.

Thus, this study expanded Byram’s factors of ICC—originally knowledge, attitude, awareness, and skills—into six factors: native cultural knowledge, foreign cultural knowledge, attitude, awareness, resilience skills, and pragmatic skills. This created a framework for the ICC of government leading officials, which was used to design the survey questionnaire, establish the indicator system, and analyze the results.

1. Native Cultural Knowledge: This included familiarity with the historical development, political stance, values, laws, regulations, and moral norms of one’s own culture.
2. Foreign Cultural Knowledge: This included knowledge of the social habits, values, and etiquette of the target culture, as well as an understanding of the historical and current relationships between one’s own culture and the target culture. Such knowledge directly influenced the ability of government leading officials to handle foreign affairs and their attitudes toward the target culture.
3. Intercultural Communication Awareness: This emphasized a critical cultural awareness, focusing on government leading officials’ ability to interpret, analyze, understand, and evaluated events and situations from other cultures while connecting them to their own cultural context.
4. Intercultural Communication Attitude: This included curiosity, openness, and the suspension of disbelief toward other cultures, as well as confidence in one’s own culture. It reflected government leading officials’ positive or negative reactions to intercultural communication, such as acceptance or rejection, agreement or opposition.
5. Resilience Skills for Intercultural Communication: This referred to the ability to interpret cultural phenomena of other cultures and relate them to one’s own cultural context. It also included the ability to adapt to new cultures and apply acquired knowledge to manage cultural differences.
6. Pragmatic Skills for Intercultural Communication: This involved the ability to correctly use a language shared by both parties, appropriately employ gestures, facial expressions, and body language to enhance communication effectiveness, and use strategies such as semantic substitution, semantic switching, and language transfer to compensate for language deficiencies.

The questionnaire (see Appendix) consisted of two parts: basic information and survey items based on factors of ICC. The first part collected the demographic variables of the participants (gender, age, educational status, type of organization, position level, whether they are in charge of foreign affairs, working years, highest level of foreign language, whether they participate in intercultural activities, frequency of encounters with foreigners, and experience of going abroad). The second part (including 31 items) was designed to investigate the participants’ intercultural communication status and factors of ICC. Items were rated on a 5-point Likert scale ranging from 1 (“Strongly disagree”) to 5 (“Strongly agree”).

#### 2.2.2 Theoretical model and path analysis

This study designed a framework aligned with the ICC model for Chinese government leading officials and referenced variables influencing ICC from existing literature to construct the theoretical analysis framework. The framework comprehensively examined how multiple conditions may interact and align to influence the ICC of government leading officials, empirically exploring diverse pathways for enhancing the ICC of this group.

This study strictly followed the logical framework of “identifying problems—proposing hypotheses—testing hypotheses—drawing conclusions.” First, based on the current development status of the Belt and Road Initiative, the research hypotheses were proposed. Second, a research hypothesis model was constructed, encompassing six factors of ICC. Third, the proposed hypotheses were tested through empirical analysis. The empirical analysis included: (1) Descriptive statistical analysis was used to examine the mean values of ICC factors, laying the groundwork for targeted recommendations. (2) Hierarchical regression analysis was employed to examine the relationships among native cultural knowledge, foreign cultural knowledge, intercultural communication attitude, intercultural communication awareness, and resilience skills for intercultural communication. (3) Independent samples *t*-tests and one-way ANOVA were employed to examine the difference of influence of demographic variables on ICC factors. This helped identify control variables for subsequent hierarchical regression analysis. (4) Hierarchical regression analysis was utilized to test the contribution of the highest foreign language score, participation in training, education status, and frequency of contact with foreigners on the ICC of government leading officials. Finally, the results of the above data analysis were summarized to draw research conclusions. Based on these findings, recommendations were proposed to contribute to the enhancement of ICC of government leading officials.

#### 2.2.3 Data collection and analysis

After data collection, the datasets were organized, and invalid data were excluded, resulting in 578 valid datasets. Subsequently, SPSS21.0 was utilized to perform descriptive analysis, reliability, and validity analysis (including exploratory factor analysis (EFA) and confirmatory factor analysis (CFA)), correlation analysis, difference analysis, and hierarchical regression analysis. AMOS 26.0 was employed to conduct CFA to validate the structure of the data.

## 3 Research results

### 3.1 Descriptive analysis

In the descriptive statistics of the participants’ ICC (as shown in Table 1), the results of the knowledge, attitude, awareness, and skills of the government leading officials in intercultural situations are presented. It can be seen that among the factors of ICC of government leading officials, the mean value of intercultural communication awareness (*M* = 3.9) was the highest, indicating that government leading officials were capable of distinguishing the differences and similarities between different cultures. The mean value of native cultural knowledge (*M* = 3.8) was high. The mean value of intercultural communication attitude was 3.7, indicating that government leading officials had a positive attitude toward intercultural communication. The mean value of pragmatic skills for intercultural communication (*M* = 2.5) was lower than resilience skills for intercultural communication (*M* = 3.4), which indicated that government leading officials do not have enough skills to use foreign languages.

**Table 1:** Descriptive statistics of survey samples (*N* = 578).

Factors of ICC of government leading officials	Minimal value	Maximum value	Mode (statistics)	Mean value	Standard deviation
Native cultural knowledge	1.7	5.0	4.0	3.8	0.66
Foreign cultural knowledge	1.0	4.7	2.6	2.5	0.63
Intercultural communication attitude	2.0	5.0	3.7	3.7	0.52
Intercultural communication awareness	1.5	5.0	4.0	3.9	0.58
Resilience skills for intercultural communication	1.0	5.0	3.0	3.4	0.68
Pragmatic skills for intercultural communication	1.0	5.0	2.0	2.5	0.98

### 3.2 Reliability and validity analysis

#### 3.2.1 Reliability analysis

To enhance the credibility of the reliability test, this study employed homogeneity reliability to examine all the data. Homogeneity reliability is represented by the internal consistency coefficient (Cronbach’s Alpha, denoted as *α*). As shown in Table 2, the α coefficients for the factors of the ICC of government leading officials range from 0.713 to 0.897, while the overall scale’s α coefficient is 0.908. These results indicated that the scale and its factors exhibited good internal consistency and stability.

**Table 2:** Reliability analysis of the ICC scale of government leading officials (*N* = 578).

	α coefficients	Indicators number
Foreign cultural knowledge	0.879	7
Native cultural knowledge	0.876	3
Intercultural communication attitude	0.713	7
Intercultural communication awareness	0.893	8
Resilience skills for intercultural communication	0.870	4
Pragmatic skills for intercultural communication	0.775	2
Total scale	0.908	31

#### 3.2.2 Validity analysis

**Figure 1:**
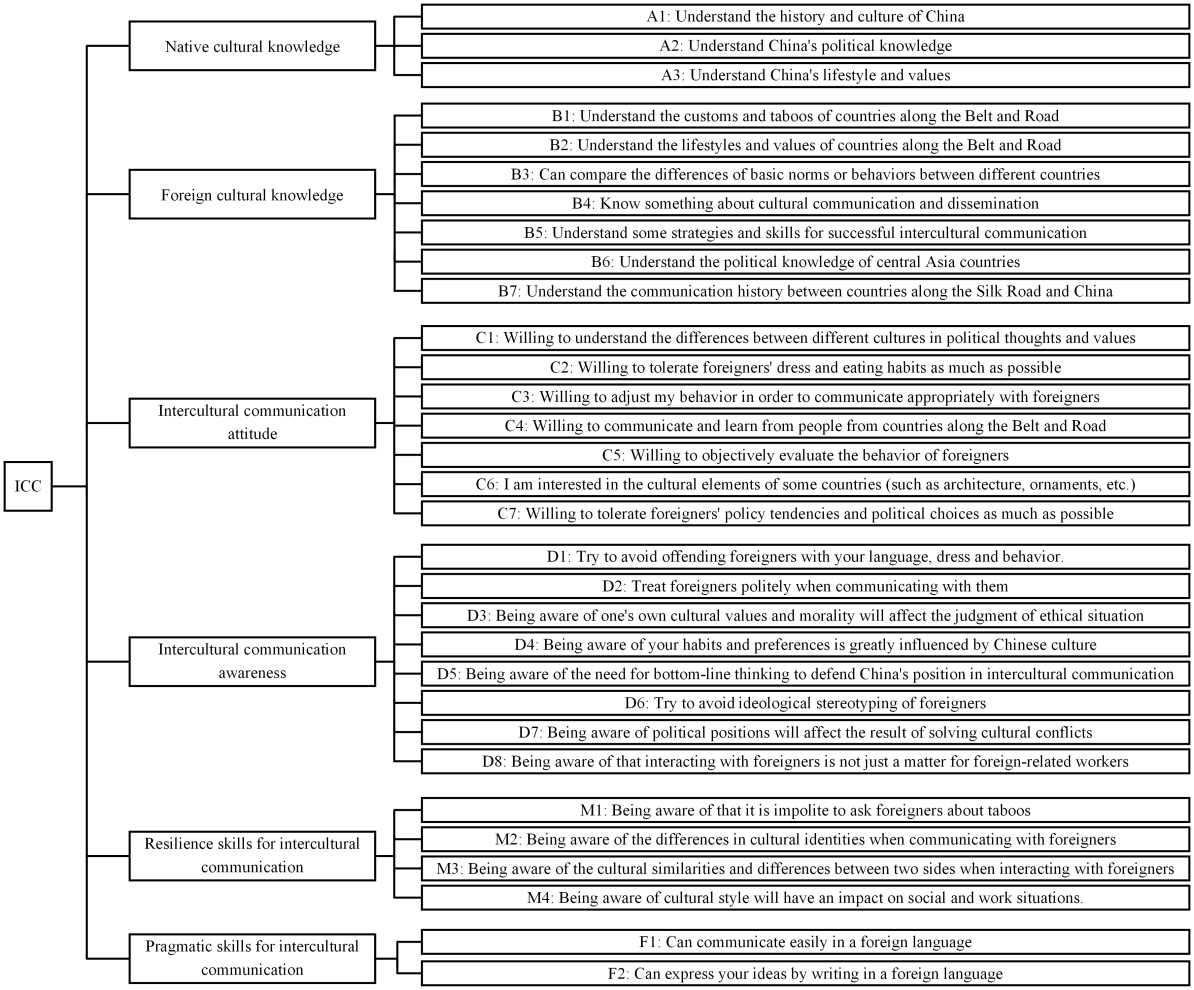
Comprehensive measurement framework of the scale.

The validity analysis results showed that the KMO value was 0.900, which was greater than 0.7. The significance of the Bartlett sphericity test was less than 0.001, indicating that the data were suitable for factor analysis. Principal component analysis was applied to conduct exploratory factor analysis on the sample data. Using the varimax orthogonal rotation method, the rotated factor loading matrix was obtained. The number of factors was determined based on eigenvalues greater than 1, ultimately extracting 6 common factors with a cumulative contribution rate of 65.662%. These 6 common factors can establish a measurement system for the ICC of government leading officials. The system comprised 6 first-level evaluation indicators and 31 s-level evaluation indicators. The detailed structure is illustrated in Figure 1.

**Figure 2:**
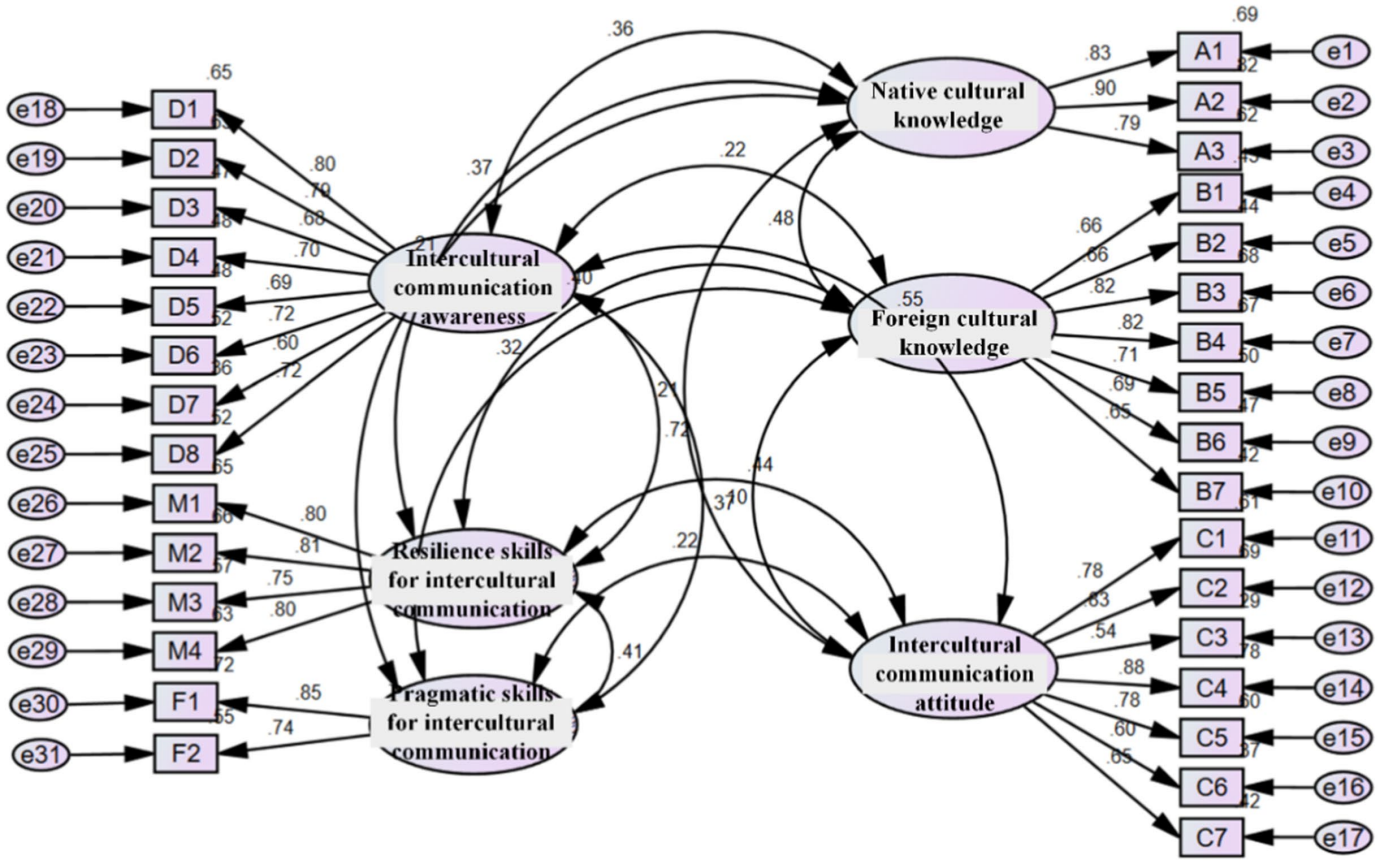
Confirmatory factor analysis model diagram.

Confirmatory factor analysis model (as illustrated in Figure 2) was constructed to further assess the structural validity of the scale. The results of fit indices for confirmatory factor analysis are listed in Table 3. These fit indices were close to the recommended thresholds, indicating that the scale had good structural validity and that the structural equation measurement model was reasonably constructed.

**Table 3:** Results of fit indices for confirmatory factor analysis.

Indices	χ^2^/df	CFI	GFI	AGFI	TLI	NFI	RMSEA
Results	4.422	0.861	0.809	0.774	0.846	0.829	0.077

### 3.3 Correlation analysis

After conducting the aforementioned tests on the data, a Pearson correlation analysis was performed on the six factors to preliminarily examine the hypothesized relationships. As shown in Table 4, it can be seen that the correlation between “intercultural communication awareness” and “resilience skills for intercultural communication” was the highest, reaching 0.644, indicating that there was a very high positive correlation between intercultural awareness and skills. The correlation between “native cultural knowledge” and “foreign cultural knowledge” was 0.425, indicating that the correlation between the two factors of government leading officials’ intercultural knowledge was high. Similarly, the correlation between “knowledge of foreign culture” and “resilience skills for intercultural communication” was 0.386, which shows that the more government leading officials know about the culture of the foreign country, the more they can apply it to intercultural communication. In conclusion, this set of data shows that there was a significant correlation between multiple factors of ICC of government leading officials, thus confirming from the data level the previous argument about the mutual influence and interaction among the six factors of ICC.

**Table 4:** Correlation coefficient of ICC in each factor (*N* = 578).

	Native cultural knowledge	Foreign cultural knowledge	Intercultural communication attitude	Intercultural communication awareness	Resilience skills for intercultural communication	Pragmatic skills for intercultural communication
Native cultural knowledge	1					
Foreign cultural knowledge	0.425**	1				
Intercultural communication attitude	0.190**	0.130**	1			
Intercultural communication awareness	0.355**	0.226**	0.491**	1		
Resilience skills for intercultural communication	0.334**	0.386**	0.374**	0.644**	1	
Pragmatic skills for intercultural communication	0.179**	0.293**	0.165**	0.299**	0.338**	1

**Significant correlation at 0.01 level (bilateral).

### 3.4 Hypothesis testing

Since the correlation between “intercultural communication awareness” and “resilience skills for intercultural communication” among government leading officials reached the highest level (0.644), we conducted regression analysis based on the regression equation with “intercultural communication awareness” as the independent variable. The results are presented in Table 5.

**Table 5:** Hierarchical regression analysis of resilience skills for intercultural communication.

Variables	Model 1	Model 2	Model 3	Model 4
Intercultural communication awareness	0.644***	0.586***	0.561***	0.515***
Foreign cultural knowledge		0.254***	0.229***	0.229***
Pragmatic skills for intercultural communication			0.103**	0.097**
Intercultural communication attitude				0.099**
Adjusted R^2^	0.413	0.473	0.482	0.488
*F*	407.199***	260.339***	179.666***	138.631***

*p < 0.05, **p < 0.01, ***p < 0.001.

The analysis revealed that: Intercultural communication awareness (*β* = 0.515, *p* < 0.001), foreign cultural knowledge (*β* = 0.229, *p* < 0.001), intercultural communication attitude (*β* = 0.099, *p* = 0.004), pragmatic skills for intercultural communication (*β* = 0.097, *p* = 0.003) were significantly and positively predicted resilience skills for intercultural communication. However, native cultural knowledge (*β* = 0.022, *p* = 0.520) showed no predictive effect. Collectively, these variables explained 48.80% of the variance in resilience skills for intercultural communication. The model demonstrated that Hypothesis H1 was partially supported. Positive correlations were observed between (1) intercultural communication awareness, (2) foreign cultural knowledge, (3) intercultural communication attitude, and (4) pragmatic skills for intercultural communication. Among these factors, intercultural communication awareness exhibited the strongest influence on resilience skills.

### 3.5 Variables that influencing ICC

ICC of government leading officials were inevitably affected by their own learning experience and communication experience. All of the demographic variables were subjected to independent samples *t*-test or one-way ANOVA. The results showed that the highest foreign language score, whether they participate in intercultural activities, educational status, and frequency of encounters with foreigners had a great influence on ICC.

Table 6 shows that government leading officials who have passed foreign language level exams are more competent in intercultural communication than those without any foreign language certificates. If the significance level was set at 0.05, there was a significant difference between with or without a foreign language certificate in terms of factors of ICC except native cultural knowledge.

**Table 6:** Independent sample *T*-test with or without proof of foreign language achievement.

With or without a foreign language certificate?	Foreign cultural knowledge	Native cultural knowledge	Intercultural communication attitude	Intercultural communication awareness	Resilience skills for intercultural communication	Pragmatic skills for intercultural communication
With	2.52 ± 0.59	3.86 ± 0.64	3.76 ± 0.49	3.97 ± 0.67	3.53 ± 0.77	3.04 ± 0.92
Without	2.48 ± 0.64	3.70 ± 0.66	3.65 ± 0.55	3.81 ± 0.69	3.27 ± 0.83	2.19 ± 0.88
*t*	0.602	2.767^**^	2.427^*^	2.707^**^	3.679^***^	10.949^***^
*p*	0.548	0.006	0.016	0.007	0.000	0.000

*p < 0.05, **p < 0.01, ***p < 0.001.

Table 7 shows that intercultural communication training will have a positive impact on the five factors of ICC of government leading officials. The mean values of all factors are higher for government leading officials with intercultural communication training experience than for those without such experience.

**Table 7:** Independent sample test for participation in intercultural communication training or not.

Participation in training	Foreign cultural knowledge	Native cultural knowledge	Intercultural communication attitude	Intercultural communication awareness	Resilience skills for intercultural communication	Pragmatic skills for intercultural communication
Yes	2.61 ± 0.63	3.90 ± 0.72	3.70 ± 0.58	4.07 ± 0.67	3.62 ± 0.84	2.88 ± 1.0
No	2.47 ± 0.62	3.73 ± 0.65	3.69 ± 0.52	3.83 ± 0.69	3.32 ± 0.80	2.42 ± 0.96
*t*	1.890*	2.186^*^	0.265	3.078^**^	3.152^**^	4.108^***^
*p*	0.049	0.029	0.791	0.002	0.002	0.000

*p < 0.05, **p < 0.01, ***p < 0.001.

Table 8 shows that education status is positively related to ICC. Government leading officials with postgraduate education or above show rich native cultural knowledge and have stronger intercultural communication awareness, as well as more intercultural communication skills. Government leading officials with college degrees and below had the lowest level of ICC.

**Table 8:** Summary table of one-way ANOVA for education status.

Education status	Foreign cultural knowledge	Native cultural knowledge	Intercultural communication attitude	Intercultural communication awareness	Resilience skills for intercultural communication	Pragmatic skills for intercultural communication
College degree and below	2.46 ± 0.71	3.69 ± 0.77	3.73 ± 0.62	3.71 ± 0.77	3.23 ± 1.0	2.04 ± 0.94
Undergraduate degree	2.49 ± 0.62	3.73 ± 0.65	3.66 ± 0.52	3.83 ± 0.69	3.33 ± 0.79	2.44 ± 0.92
Postgraduate degree and above	2.53 ± 0.61	3.89 ± 0.61	3.75 ± 0.52	4.06 ± 0.61	3.54 ± 0.76	2.92 ± 1.0
*F*	0.324	3.183*	1.451	7.232**	3.905*	20.579***
*p*	0.723	0.042	0.235	0.001	0 0.021	0.000

*p < 0.05, **p < 0.01, ***p < 0.001.

Table 9 shows that the frequency of contact with foreigners is related to the six factors of ICC of government leading officials. It can be seen that the higher the frequency of contact with foreigners, the higher the mean value of each factor of ICC, and vice versa. This indicated that ICC of government leading officials is deeply affected by the experience of intercultural interaction.

**Table 9:** Summary of one-way ANOVA for frequency of contact with foreigners.

Frequency of contact with foreigners	Foreign cultural knowledge	Native cultural knowledge	Intercultural communication attitude	Intercultural communication awareness	Resilience skills for intercultural communication	Pragmatic skills for intercultural communication
Often	2.57 ± 0.00	4.00 ± 0.00	3.93 ± 0.30	4.00 ± 0.00	3.63 ± 0.53	3.00 ± 0.00
Sometimes	2.74 ± 0.45	3.55 ± 0.80	3.96 ± 0.54	4.27 ± 0.37	3.66 ± 0.78	3.32 ± 1.20
Occasionally	2.65 ± 0.50	3.91 ± 0.62	3.69 ± 0.49	4.06 ± 0.58	3.55 ± 0.76	2.82 ± 1.96
Seldom	2.61 ± 0.63	3.85 ± 0.69	3.78 ± 0.51	3.91 ± 0.69	3.54 ± 0.78	2.75 ± 1.01
Rarely	2.38 ± 0.63	3.68 ± 0.63	3.62 ± 0.54	3.78 ± 0.71	3.21 ± 0.82	2.24 ± 0.88
*F*	5.78^***^	3.43^**^	3.89^**^	3.61^***^	6.62^**^	14.30^***^
*p*	0.000	0.009	0.004	0.006	0.000	0.000

*p < 0.05, **p < 0.01, ***p < 0.001.

### 3.6 Influence variables of ICC

This study employed hierarchical regression analysis to explore the variables influencing the ICC of government leading officials, as listed in Table 10. The analysis process constructed four stepwise regression models (M1 to M4), aiming to gradually incorporate different independent variables and examine their individual and combined effects on ICC.

**Table 10:** Hierarchical regression analysis of ICC of government leading officials.

Variables	Model 1	Model 2	Model 3	Model 4
Highest foreign language score	0.194***	0.181***	0.154***	0.131**
Participation in training		0.123**	0.110**	0.039
Education status			0.085	0.078
Frequency of contact with foreigners				0.191***
Adjusted R^2^	0.036	0.049	0.054	0.083
*F*	22.588***	15.973***	11.975***	14.001***

*p < 0.05, **p < 0.01, ***p < 0.001.

In the first model, only the highest foreign language score was included as an independent variable. The results showed that the highest foreign language score had a significant positive impact on ICC (*β* = 0.194, *p* < 0.001), indicating that better foreign language performance was associated with stronger ICC among government leading officials. The model demonstrated a good overall fit, with an adjusted R^2^ of 0.036 and an *F*-value of 22.588 (*p* < 0.001), explaining 3.6% of the variance in ICC.

In Model 2, the variable of whether they had participated in intercultural training was added. The highest foreign language score remained significant (*β* = 0.181, *p* < 0.001), and whether they had participated in intercultural training also had a significant positive impact (*β* = 0.123, *p* = 0.003). This suggested that participation in intercultural activities can further enhance the ICC of government leading officials. The model’s explanatory ability improved, with an adjusted R^2^ of 0.049 and an *F*-value of 15.973 (*p* < 0.001), explaining 4.9% of the variance in ICC.

Model 3 further incorporated the variable education status. The highest foreign language score (*β* = 0.154, *p* < 0.001) and whether they had participated in intercultural training (*β* = 0.110, *p* = 0.008) remained significant, while the influence of education status approached significance (*β* = 0.085, *p* = 0.051). Although educational background did not fully reach significance, it may still have some impact on ICC. The model’s explanatory ability continued to increase, with an adjusted R^2^ of 0.054 and an *F*-value of 11.975 (*p* < 0.001), explaining 5.4% of the variance in ICC.

In the fourth model, the variable frequency of contact with foreigners was added. The highest foreign language score (*β* = 0.131, *p* = 0.002) still had a significant positive impact on ICC, while whether they had participated in intercultural training became insignificant (*β* = 0.039, *p* = 0.372). The frequency of contact with foreigners domestically showed a significant positive impact on ICC (*β* = 0.191, *p* < 0.001). This indicated that the more frequently government leading officials interact with foreigners, the stronger their ICC tended to be. This phenomenon may stem from frequent intercultural contact enhancing their cultural confidence, enabling them to communicate and interact more comfortably and confidently in multicultural environments. Model 4 had the strongest explanatory ability, with an adjusted R^2^ of 0.083 and an *F*-value of 14.001 (*p* < 0.001), explaining 8.3% of the variance in ICC.

Combining the results of the four models, the highest foreign language score had a stable and significant positive impact on the ICC of government leading officials, indicating that strong foreign language proficiency was an important foundation for intercultural communication. Lysiuchenko et al. (2021) proved that ICC was closely linked to the development of students’ linguistic skills. High-stakes English language proficiency tests to serve increasingly critical gate-keeping roles for people seeking admission to tertiary institutions (Meyer, 2017). Whether they had participated in intercultural training was significant in Models 2 and 3 but was replaced by frequency of contact with foreigners in Model 4, which had a more direct impact on ICC. Although educational status approached significance in Model 3, its overall impact was limited.

## 4 Discussion

### 4.1 The current situation of ICC of government leading officials of Xi’an

Organizations such as governments and companies need global leaders who can interact in different cultural environments (Armstrong, 2019; Ngai and Singh, 2018). Identifying and equipping these future leaders with ICC is a prerequisite for governments and companies to be successful around the world (Lawrence, 2015). Influenced by the traditional Chinese concept of “harmony in diversity,” Government leading officials of Xi’an exhibit an awareness of seeking common ground while reserving differences when faced with cultural diversity. Combined with traditional Chinese philosophical ideas, Chen and An (2009) proposed an ICC model of Chinese leadership containing three dimensions including self-cultivation, context profundity, and action dexterity. The results showed that Chinese people had unique intercultural communication skills. A study (Deddy Mulyana Agustina, 2015) showed that Chinese people were good at expressing their self-presentation by employing various verbal and nonverbal tactics to adjust themselves to interpersonal, group, and business situations during communication with Malays.

In the context of the Belt and Road Initiative, government leading officials in Xi’an operated in an environment characterized by regional collaboration, economic and trade exchanges, and cultural interactions. Their intercultural communication awareness and intercultural communication attitude scores were notably high, reflecting a deep recognition of the need to adapt culturally and behaviorally. However, while these government leading officials exhibited a strong willingness for intercultural communication, they often struggled to fully appreciate the challenges of cultural adaptation in specific intercultural situations. They found it difficult to empathize with the cultural circumstances of others from different cultural backgrounds. This lack of emotional resonance between people from different cultures often lead to cultural biases and misinterpretations.

From the perspective of the external cultural environment, the relatively inactive nature of their living and working environments had resulted in a weaker intercultural emotional connection among some government leading officials. The survey results of this study showed that 92.4% of government leading officials perceive that Xi’an had become more open than before under the Belt and Road Initiative. However, their social interactions were largely confined to colleagues, relatives, and friends within a familiar circle. For thousands of years, China’s agrarian-based economy fostered a stable living environment, with a strong emphasis on settling in one place and low cross-border mobility.

Intercultural communication skills were important. Effective communication was a critical skill of the leader (Szczepański and Kanik, 2021). A leader should develop reasoning and emotional intelligence as skills, in this way, the employees were encouraged to work harder for that leader (Chatman et al., 2020). The results of the study of intercultural communication skills of Indonesian leader (Gathmyr, 2021) showed that intercultural communication skills were indispensable qualities of political party cadres, who will run various regions in Indonesia with cultural diversity. This study also found that Indonesian leaders lack systematic intercultural communication knowledge and skills, as well as the awareness and ability to use intercultural communication skills to resolve conflicts. They were also lacking in intercultural communication training. Through intercultural communication training, future leaders can improve the quality of their leadership. This was similar to the conclusion of this study that government leading officials need intercultural communication training.

Gudykunst (1988, 1998) created the Anxiety and Uncertainty Management (AUM) theory. The theory suggested that the effectiveness of communication was dependent on the ability to reduce misunderstandings. Too much anxiety or too much uncertainty reduced the ability and confidence to process information. This study also found that government leading officials with lower frequencies of interaction with foreigners tend to experience anxiety and discomfort when faced with unfamiliar intercultural communication situations.

It was evident that government leading officials in China have limited opportunities to interact with foreigners, leading to insufficient confidence in intercultural communication. As a result, they often struggled to empathize with individuals from different cultural backgrounds. From the perspective of intrinsic cultural values, the diversity and complexity of cultural values made it difficult for Chinese government leading officials to achieve the desired outcomes in intercultural communication. We specifically examined how the majority group members in the local cultural environment perceive foreign minority cultures. Foreign cultures, belonging to different cultural value systems, are characterized by diversity and complexity. Huntington (2011) categorized world civilizations into Christian, Orthodox, Islamic, and others. He argued that as globalization advances, differences in values and identities among civilizations may intensify, becoming a significant source of conflict.

The survey findings revealed that in the context of the Belt and Road Initiative, Chinese government leading officials welcome cross-border populations coming to China for business, trade, tourism, and other activities. However, they found it challenging to quickly understand the values and behavioral habits of their intercultural communication counterparts. For example, it was generally believed that Eastern cultures emphasize emotionality, intuition, and collectivism, while Western cultures prioritized individualism, achievement orientation, rationality, and logic. The differences in cultural values were seen as an insurmountable gap for cultural exchange and information transfer between individuals or groups. Despite the willingness of individuals from different cultural backgrounds to communicate, they often misinterpreted each other’s cultures, which became the greatest obstacle to intercultural communication.

### 4.2 Strategies for improving ICC of government leading officials

Since the Belt and Road was proposed, China’s ICC has faced great challenges and at the same time ushered in new opportunities (Xiaoni, 2019). On the one hand, intercultural communication can enhance political mutual trust, and on the other hand, it can help eliminate prejudices, break down barriers, and promote integration (Xiaoyu, 2021). Intercultural communication enabled Chinese products and enterprises to successfully “go out” and realized economic integration with Belt and Road participating countries (Lu and Yu, 2015).

Language skills were considered to be an important variable influencing ICC, and the language barrier is usually an important obstacle faced by government leading officials of China in intercultural communication. Studies have shown that the current English language teaching in China, which was based on university English teaching, cannot meet the needs of economic cooperation with countries along the Belt and Road (Zhenhuan and Xia, 2016). The cultivation of foreign language talents for intercultural communication in the context of Belt and Road required a change in teaching concepts and the cultivation of two-way cultural communication awareness (Liu, 2020). Universities and training institutions needed to provide relevant intercultural communication courses and carry out intercultural communication practices.

The required language skills of government leading officials should be adjusted according to different regions. For example, in regions with a low degree of openness, the foreign language skills of government leading officials should be regarded as a tool for understanding foreign cultures, which can be used to strengthen government leading officials’ understanding of the national policy of other countries and the international environment. In regions with a higher degree of openness, foreign language skills should be focused on communicating without barriers. The Academy of Governance should set up an “English Application Enhancement” training course, which teached business etiquette and English expression that was applicable to the actual work of government leading officials. In addition, the positions and duties of government leading officials should also be incorporated into the foreign language education strategy. For example, professional foreign language courses should be offered to meet the foreign language needs of government leading officials involved in foreign affairs work.

In addition to language, non-verbal symbols such as eyes, facial expressions, body postures, and dress codes can effectively compensate for deficiencies in verbal expression in communicating with people from different cultural backgrounds. This inspired government leading officials to make more reasonable use of non-verbal behaviors to understand the feelings and to compensate for the lack of verbal expression.

Professional training is an important way to rapidly improve ICC. Both quantitative and qualitative evaluations showed that adopting online teaching may serve as an effective way to develop students’ ICC (Shen, 2021). A study showed that the intercultural communication training increased the level of ICC (Hastjarjo and Nuryana, 2018). Party schools and academies of governance at all levels should adhere to the principle of categorization in their training of government leading officials in intercultural communication knowledge. The training contents should be applicable to the needs of different groups of government leading officials. For regions with a high degree of internationalization, as well as for foreign affairs departments, it is necessary to concentrate on in-depth, targeted training in communication and conflict-management skills, in accordance with the needs of the work. Seminars such as the construction of Belt and Road, improving the overall quality of government leading officials in foreign affairs should be organized regularly. The purpose of these trainings is to help government leading officials to gain a better understanding of the religious, laws, customs, and cultural knowledge of the countries along the Belt and Road.

We can also adopt more dynamic teaching methods to enhance the ICC of government leading officials. Due to limited intercultural contacts and scenes in inland regions, government leading officials in these regions often struggle to understand and apply intercultural communication knowledge. Therefore, training institutions can explore teaching methods such as intercultural scenario simulations, case studies, and identity immersion to create interactive scenarios for cultural exchange and deepen the emotional engagement of government leading officials. For example, we can use on-site observations and role-switching activities to conduct practical training in transnational project sites or multinational enterprise workshops, enriching teaching formats and providing government leading officials with experiential learning opportunities. Additionally, we can leverage the internet to develop a remote education platform for the training of government leading officials. Through the “Internet + Training” approach, government leading officials can overcome time and space constraints, enabling interaction among experts, instructors, and officials. This will allow for more effective utilization of abundant online educational resources and improve the knowledge and competencies of government leading officials in intercultural communication.

It is beneficial for Chinese government leading officials to have more opportunities to touch foreign cultures through social life. Kim (2015) argued that differences created a degree of stress on intercultural communication, which in turn created desynchronization. This desynchronization provided an opportunity to pursue synchronization in intercultural communication. For government leading officials in inland cities who have been in a relatively closed cultural environment, their ICC can be improved through daily life. For example, eating foreign food, enjoying foreign movies and entertainment, and traveling, studying, and working abroad are desynchronizations. These activities can promote government leading officials to perceive cultural differences in informal communication. However, it is difficult to cultivate ICC of government leading officials through social life in a targeted and systematic way. Therefore, it is necessary to consciously borrow social experiences for intercultural training. Nardon (2017) borrowed from transformative learning theory and explored the important role of context in intercultural communication: the creation of effective contexts contributed to a deeper understanding of the intercultural learning process and promoted ICC.

For example, thematic activities should be used to inspire participants, such as discussing their favorite foreign movies and foreign football stars, how to look at the global proliferation of McDonald’s, or how to conduct business negotiations with foreign businessmen. With the help of foreign cultures in social life, government leading officials can be consciously guided to interpret some issues from an intercultural perspective, to enhance intercultural awareness. Of course, government leading officials can also be encouraged to watch intercultural-related programs or even participate in various intercultural activities to realize intercultural situational education.

Government leading officials may also be selected to receive academic education abroad to learn from the governance experience of other countries, which is more conducive to expanding their international vision. According to the needs of the work, some government leading officials should be selected to go abroad for training and further study, to cultivate government leading officials with a global vision and the competence to innovate.

According to Berry (2008) Acculturation Theory, individuals can maintain both a preference for their native culture and a tendency to interact with other cultural groups. Government leading officials of Xi’an are encouraged to establish cooperative relations with universities, research institutes, and professional training institutions in countries along the Belt and Road Initiative. Government leading officials of Xi’an can exchange China’s development achievements and experiences in poverty reduction, industrialization, infrastructure, industrial parks, and other fields in the “the belt and road initiative” seminar, and jointly explore development paths suitable for their own national conditions with leading officials of other countries. Promoting educational cooperation in Xi’an under the Belt and Road Initiative can, on the one hand, broaden the horizons of government leading officials in inland cities and advance the development of Chinese culture. On the other hand, it can facilitate broader cultural exchanges and promote interaction among the world’s diverse cultures. When Chinese government leading officials engage in overseas learning, it also contributes to the study of intercultural communication from a communication studies perspective. This perspective emphasizes the process and outcomes of how cultures are accepted after leaving their original context.

Giles and Noels (1997) introduced the Communication Accommodation Theory (CAT). The theory suggested that in different cultural situations, people used language to adjust their behaviors as little as possible to adapt to the main culture, thus narrowing or widening the distance between cultures. Overseas academic education for Chinese government leading officials is a process of narrowing the cultural distance between their native culture and the target culture, as well as an application and validation of the Communication Accommodation Theory.

### 4.3 Limitations

This study has certain limitations. First, we did not explore the intrinsic mechanisms behind the ICC of Chinese government leading officials, such as the positive role of cultural sensitivity in ICC or the mediating role of cultural confidence between intercultural contact and ICC. To expand on these aspects, we need to adjust the structure of the survey questionnaire by adding measurement scales for cultural sensitivity and cultural confidence. Additionally, we can propose and test new hypotheses. We can incorporate additional potential influencing variables, such as international work experience and cultural sensitivity, to provide a more comprehensive understanding of the formation mechanisms of ICC among government leading officials. Longitudinal research designs could be adopted to track the development of ICC among government leading officials at different stages, revealing its dynamic progression. These studies will help provide more targeted and effective strategies and recommendations for cultivating the ICC of government leading officials.

Second, the sample selected for this study primarily consisted of government leading officials from Xi’an. Due to limitations in resources, the findings reflect the current situation in Xi’an or Shaanxi Province and may not be generalizable to government leading officials across China. In further research, we plan to select representative cities from both the more economically developed eastern regions and the less developed central and western regions of China to collect a broader sample for comparative analysis. The analysis of ICC among Chinese government leading officials is an ongoing research endeavor that requires deeper exploration based on the existing findings.

## 5 Conclusion

This study drew on Byram’s Intercultural Communication Competence model and integrated the practical context of the Belt and Road Initiative to construct a tailored ICC model for Chinese government leading officials. A survey questionnaire was designed, and the status and influencing variables of ICC among government leading officials were analyzed using Xi’an, as a case study. Possible measures to enhance ICC were also proposed. The research achievements were reflected in two main aspects.

In terms of theoretical contributions, this study adopted the widely recognized factors of knowledge, attitude, awareness, and skills to evaluate the ICC of Chinese government leading officials, while redefining these factors. We placed greater emphasis on how intercultural communicators in non-English-speaking regions overcome language barriers. Language barriers in intercultural communication can lead to psychological tension and discomfort, hindering the effectiveness of intercultural interactions. This focus optimized the existing literature on the structure of ICC, making it applicable to broader regions and more diverse populations. Additionally, the ICC framework constructed in this study emphasized bidirectional interaction between one’s own culture and foreign cultures, rather than a one-way cultural output from one’s own culture to foreign cultures. This perspective also contributed positively to improving cultural communication paradigms.

In terms of practical applications, this study found that Chinese government leading officials highly respected the uniqueness of the target culture in intercultural communication and recognized the role of cultural exchange in the Belt and Road Initiative. Government leading officials in Xi’an actively embraced the cultures of countries and ethnic groups along the Belt and Road, thereby enhancing the city’s openness and internationalization. However, their insufficient understanding of the target culture, such as holiday customs and religious taboos in Belt and Road countries, may lead to cultural misunderstandings. The lack of resilience skills and pragmatic skills in intercultural communication among Chinese government leading officials also posed risks to effective intercultural interactions. The survey revealed that variables such as the highest foreign language score, participation in training, education status, and frequency of contact with foreigners significantly influenced ICC. Therefore, we can consider offering professional English training courses to meet the language needs of government leading officials involved in foreign affairs. We can also organize thematic seminars on the Belt and Road Initiative and platforms such as the “Young Leaders Training Program” to provide government leading officials with multicultural exchange scenarios, enhancing their knowledge and adaptability in intercultural communication. Additionally, we can encourage Chinese government leading officials to actively engage with diverse cultures through activities such as international travel, studying abroad, and learning foreign languages. For government leading officials with foreign-related responsibilities, we can organize overseas training programs to broaden their international perspectives. Through interactions with diverse cultures, government leading officials will gradually develop a more objective, rational, and open attitude toward intercultural communication, facilitating cultural dissemination and exchange.

## Data Availability

The original contributions presented in the study are included in the article/Supplementary material, further inquiries can be directed to the corresponding author.
