# Supplementary material for: How to improve communication competence of government leading officials in intercultural environment?—the driving role of intercultural psychological factors
Source: Front Psychol. 2025 Apr 28;16:1544626. doi: 10.3389/fpsyg.2025.1544626 (PMC12066315; doi:10.3389/fpsyg.2025.1544626)
Supplement: Supplementary file 1 [file Table_1.DOCX]

Supplementary Material

Appendix:

**Questionnaire**

Dear Sir (Madam):

Greetings! First of all, thank you for taking time out of your busy schedule to help us complete this survey.

The purpose of this survey is to examine the current situation of intercultural communication competence (ICC) of leading officials in Inland cities of China. Your participation and answers are of great significance to objectively study this issue. This questionnaire is anonymous and will be used only for academic research with strict confidence. This questionnaire will not be used for any kind of personal performance evaluation, so please feel free to answer the questionnaire according to your own real situation and real thoughts. If you are interested in this issue, you can write to me, we will feedback the research results to you.

Instructions for filling in the questionnaire: This questionnaire is divided into two main parts, in the first part we need you to provide some basic information about yourself; in the second part you will see some statements, please tick the corresponding options by √ according to your own real situation and real thoughts.

Questionnaire No. □□□

**I. Basic information**

Please tick the corresponding options by √ according to your actual situation.

Gender: □ Male □ Female

Age: □ 30 years old and below □ 31-40 years old □ 41-50 years old □ 51 years old and above

Education status:

□ College degree and below □ Undergraduate degree □ Postgraduate degree and above

Type of organization:

□ Party and government organization □ Public institution □ State-owned enterprise

Position level: □ Section-Head level □ Division-Head level □ Bureau-Director level

Are you in charge of **foreign affairs (services)** in your organization: □ Yes □ No

Working years: □ less than 5 years □ 5-10 years □ 11-20 years □ more than 20 years.

Your best score in a foreign language is:

□ No foreign language level certificate obtained

□ College English Test: CET-4 or CET-6 □ Test for English Majors: TEM-4 or TEM-8

□ Other English scores (e.g. TOEFL 90; IELTS 6, etc.)

□ Scores in **other languages** (e.g., Russian IV; French IV, etc.)

Have you participated in activities related to intercultural communication, such as international academic conferences, intercultural training, etc.: □ Yes □ No

Frequency of contact with foreigners:

□ Often □ Sometimes □ Occasionally □ Seldom □ Rarely

Have you ever gone abroad: □ Yes □ No

If you have been abroad, how long have you been abroad?

□ Within three months □ More than three months to six months

□ More than half a year to one year □ More than one year to two years □ More than two years

**Ⅱ. Survey items designed based on the ICC factors**

| Do you meet the following description? | (1) Strongly disagree | (2) Disagree | (3) Neutral | (4) Agree | (5) Strongly agree |
| --- | --- | --- | --- | --- | --- |
| Understand the history and culture of China. |  |  |  |  |  |
| Understand China's political knowledge. |  |  |  |  |  |
| Understand China's lifestyle and values. |  |  |  |  |  |
| Understand the political knowledge of central Asia countries. |  |  |  |  |  |
| Understand the communication history between countries along the Silk Road and China. |  |  |  |  |  |
| Understand the lifestyles and values of countries along the Belt and Road. |  |  |  |  |  |
| Understand the customs and taboos of countries along the Belt and Road. |  |  |  |  |  |
| Can compare the differences of basic norms or behaviors between different countries. |  |  |  |  |  |
| Know something about cultural communication and dissemination. |  |  |  |  |  |
| Understand some strategies and skills for successful intercultural communication. |  |  |  |  |  |
| Willing to understand the differences between different cultures in political thoughts and values. |  |  |  |  |  |
| Willing to tolerate foreigners' dress and eating habits as much as possible. |  |  |  |  |  |
| Willing to adjust my behavior in order to communicate appropriately with foreigners |  |  |  |  |  |
| Willing to communicate and learn from people from countries along the Belt and Road. |  |  |  |  |  |
| Willing to objectively evaluate the behavior of foreigners. |  |  |  |  |  |
| I am interested in the cultural elements of some countries (such as architecture, ornaments, etc.) |  |  |  |  |  |
| Willing to tolerate foreigners' policy tendencies and political choices as much as possible. |  |  |  |  |  |
| Try to avoid offending foreigners with your language, dress and behavior. |  |  |  |  |  |
| Treat foreigners politely when communicating with them. |  |  |  |  |  |
| Being aware of one's own cultural values and morality will affect the judgment of ethical situation. |  |  |  |  |  |
| Being aware of your habits and preferences is greatly influenced by Chinese culture. |  |  |  |  |  |
| Being aware of the need for bottom-line thinking to defend China's position in intercultural communication. |  |  |  |  |  |
| Try to avoid ideological stereotyping of foreigners. |  |  |  |  |  |
| Being aware of political positions will affect the result of solving cultural conflicts. |  |  |  |  |  |
| Being aware of that interacting with foreigners is not just a matter for foreign-related workers. |  |  |  |  |  |
| Being aware of that it is impolite to ask foreigners about taboos. |  |  |  |  |  |
| Being aware of the differences in cultural identities when communicating with foreigners. |  |  |  |  |  |
| Being aware of the cultural similarities and differences between the two sides when interacting with foreigners. |  |  |  |  |  |
| Being aware of cultural style will have an impact on social and work situations. |  |  |  |  |  |
| Can communicate easily in a foreign language |  |  |  |  |  |
| Can express your ideas by writing in a foreign language. |  |  |  |  |  |
